# Cortical bone trajectory screws for the middle-upper thorax: An anatomico-radiological study

**DOI:** 10.1097/MD.0000000000004676

**Published:** 2016-09-02

**Authors:** Sun-Ren Sheng, Jiao-Xiang Chen, Wei Chen, En-Xing Xue, Xiang-Yang Wang, Qing-An Zhu

**Affiliations:** a Nan Fang Hospital of Southern Medical University, Guangzhou; b Department of Orthopedics Surgery, Second Affiliated Hospital of Wenzhou Medical University; c Department of Radiology, Second Affiliated Hospital of Wenzhou Medical University, Wenzhou, China.

**Keywords:** anatomico-radiological study, cortical bone trajectory screws, middle-upper thorax

## Abstract

To quantify the reference data concerning the morphometrics of the middle-upper thorax to guide the placement of cortical bone trajectory (CBT) screws.

Eighty patients were studied on computed tomography (CT) scans. The reference anatomical parameters were measured. Next, 20 cadaveric specimens were implanted with CBT screws based on CT measurements. These specimens were then judged directly from the cadaveric vertebrae and X-ray.

The maximum length of the trajectory, the maximum diameter, and the cephaled angle exhibited a slight increase trend while the transverse and sagittal angles of the pedicle tended to decrease from T3 to T8. We recommend that the width of CBT screw for middle-upper thoracic spine is 5.0 mm, the length is 25 to 35 mm. The cadaveric anatomical study revealed that 5/240 screws penetrated in the medial or lateral areas, 5/240 screws penetrated in the superior or inferior pedicle wall, and 2/240 screws did not fit into the superior endplate of the pedicle.

The CBT screws are safe for the middle-upper thorax. This study provides a theoretical basis for clinical surgery.

## 1 Introduction

The pedicle screw fixation system has been widely used in spinal surgeries due to its biomechanical advantages in 3-dimensional fixations and short-segment fixations. However, complications, such as screw loosening, pullout, and breakage, often lead to the loss of surgical construct stability, particularly in patients with poor bone quality.^[1–3]^

The cortical bone trajectory (CBT)^[4]^ technique is theoretically beneficial for fixations of osteoporotic vertebrae that involve the maximization of the thread contact with the high-density bone surface. CBT screws obtain 4-point fits between the dorsal cortex at the site of insertion, the medially oriented posterior pedicle wall, the laterally oriented anterior pedicle wall, and the curvature of the vertebral body wall.^[5,6]^ Screw fixation in cortices might decrease the incidence of long-term hardware failure. The use of CBT screws is also a good rescue technique.

The pedicle instrumentation use in the thoracic spine has become increasingly popular in recent years.^[7]^ Middle-upper thoracic spinal surgeries with a pedicle screw present a challenge to orthopedic surgeons. Pedicle screws in the middle-upper thorax are associated with a greater risk of canal encroachment and vascular intrusion.^[8]^ Due to the caudocephaled path sagittally and the laterally directed path in the transverse plane, the CBT technique may have a safer profile in terms of insertion during surgery compared with the use of a pedicle screw. CBT screws that are inserted in a manner aimed toward the superior–anterior aspect of the vertebral body could provide superior rigidity during cantilever bending, which is an essential surgical technique for the sequential correction of spinal deformities through a series of compressive maneuvers.^[9]^

Some authors have reported on the application of CBT screws to the lower thoracic spine,^[10]^ lumbar spine,^[11–13]^ and sacral vertebrae.^[14]^ However, to the best of our knowledge, there are currently no reports about the introduction of CBT screws into the middle-upper thorax. How to use the CBT screws in middle-upper thorax is important, especially in patients with osteoporosis and screw loosening. The purpose of the present study was to collect morphometric measurements of middle-upper thoracic (T3–T8) CBT screws via radiological and cadaveric studies.

## 2 Materials and methods

This study was approved by the ethics committee of our institution (the Second Hospital of Wenzhou Medical College Research Ethics Committee Meeting).

### 2.1 CT scan measurements

**Figure 1:**
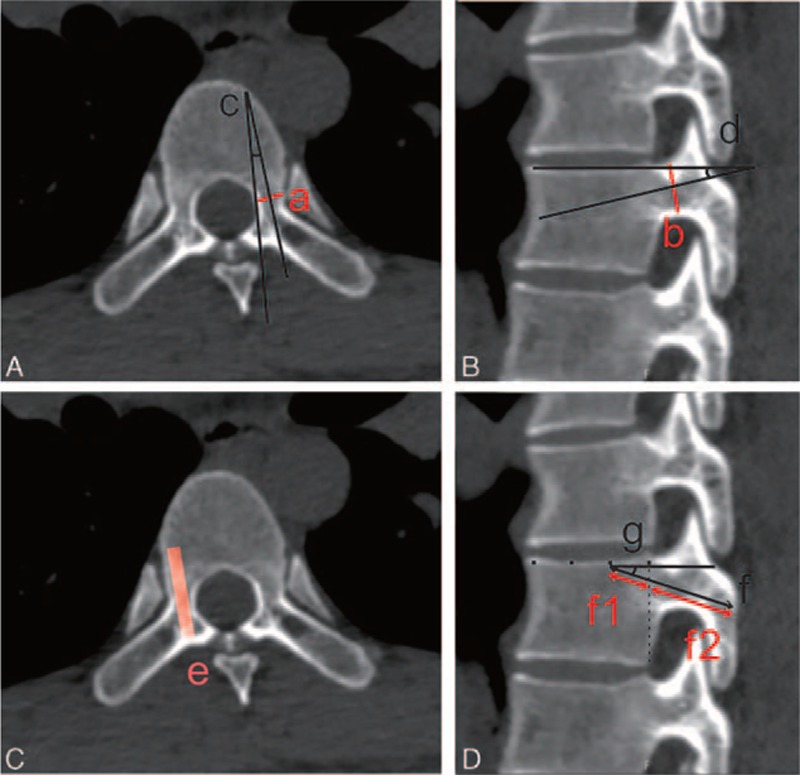
Morphometric measurement of the pedicle and trajectory. (A and B) Measurement of the pedicle. (C and D) Measurement of the trajectory. a = Pedicle width, b = pedicle height, c = the transverse angle, d = the sagittal angle, e = the maximum diameter, f = the maximum length (f1 = in-body, f2 = in-pedicle), g = the cephaled angle.

The computed tomography (CT) scans of 80 adults (47 males and 33 females; age range, 30–67 years; mean ± standard deviation [SD]: 47.0 ± 14.0 years) who presented with spinal abnormalities, such as fractures, malformations, and tumors, were excluded. A total of 480 thoracic vertebrae from T3 to T8 were observed. All CT images were measured using the postprocessing 3-dimensional reconstruction software. Three people measured the morphometric parameters of the pedicle and trajectory. The following bilateral measurements were collected from this group (Fig. 1):

1. The pedicle width (a) and height (b) at the narrowest coronal section of the pedicle.
2. The pedicle transverse angle (c), that is, the angle between the pedicle axis and a line parallel to the vertebral midline.
3. The pedicle sagittal angle (d), that is, the angle between the pedicle axis and the superior border of the vertebral body.
4. The maximum trajectory diameter which means the width of screw (e), that is, the width of the outer margin of the cortex.
5. The maximum trajectory length which means the length of screw (f), that is, the distance from the posterior aspect of the laminar cortex to the anterior aspect of the cortex of the vertebral body (in-body and in-pedicle; f1 and f2).
6. The trajectory of the cephaled angle which means the direction of screw in sagittal section (g), that is, the angle between the trajectory and the superior border of the vertebral body in the sagittal plane.

### 2.2 Cadaveric anatomical study

**Figure 2:**
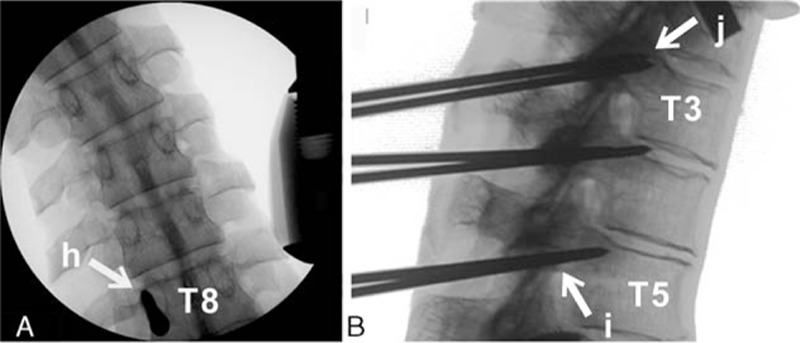
Judgment of trajectory. (A) (h) CBT screw penetrated the medial or lateral pedicle wall. (B) (i) CBT screw penetrated the superior or inferior pedicle wall, (j) CBT screw fits into the superior endplate or pedicle. CBT = cortical bone trajectory.

We also studied 20 adult cadavers (9 female and 11 male; age range, 33–66 years; mean ± SD, 46.3 ± 11.3 years) that contained the middle upper thorax (T3–T8). According to free-hand technique, the starting points of T3 to T4 and T7 to T8 were located at the junction of the midline of the superior articular process and the midline of the transverse process. The position of the T5 to T6 was taken as the intersection of the midline of the superior articular process and the 1/3 line of the superior transverse process. A K-wire was placed at the proposed entry point of the screw at an angle that was based on the results of the CT measurements and passed across the pedicle to the upper endplate of the vertebral body under computed radiography (CR) monitoring. A cannulated tap was then introduced over the K-wire. A 5.0-diameter CBT screw was inserted through the trajectory. After all of the screws were placed, anterior–posterior and lateral films were obtained. The following measurements or judgments were made side by side in this group (Fig. 2):

1. The ratio of the in-body (f1) and the in-pedicle (f2) trajectory lengths.
2. The trajectory of the cephaled angle (g), that is, the angle between the trajectory and the superior border of the vertebral body in the sagittal plane.
3. The anterior–posterior films were used to examine whether the CBT screw penetrated the medial or lateral pedicle wall (h) (we defined 3/4 screw in pedicle as place well, 1/2 screw out of pedicle as penetrated the medial or lateral pedicle wall).
4. The lateral films were used to determine whether the CBT screw penetrated the superior or inferior pedicle wall (i).
5. The lateral films were used to determine whether the CBT screw fit into the superior endplate or pedicle (j).

### 2.3 Statistical analysis

The results are presented as mean ± SD. The statistical analyses were performed with SPSS software v17.0. Comparisons of the measurements between the CT scans and the cadaveric specimens were tested using independent-samples *T* tests, and *P* values < 0.05 were considered statistically significant.

## 3 Results

### 3.1 CT scan measurements

The height of the pedicles tended to increase slightly from T3 to T8 (from 11.39 ± 0.93 mm at T3 to 12.27 ± 0.85 mm at T8). The valley of the pedicle width was 4.79 ± 0.60 mm at T4, whereas the peak was 6.80 ± 0.70 mm at T8. In contrast, the transverse and sagittal angles of the pedicle tended to decrease gradually from T3 to T8. With respect to the morphometric elements that affected the thoracic CBT, the maximum diameter and the cephaled angle played important roles. The measurements all increased gradually from T3 to T8. Similarly, the maximum lengths of the trajectories from T3 to T8 were 23.63 ± 1.96, 25.44 ± 1.88, 26.84 ± 1.82, 28.22 ± 1.42, 29.80 ± 1.69, and 31.06 ± 1.58 mm, respectively. The ratios of the in-body to the in-pedicle trajectories exhibited a similar trend. According to CT scan measurements and some publically available papers regarding pedicle anatomy in the thorax,^[15,16]^ the inside pedicle width is <5.5 mm from T3 to T8. So we recommend that the width of CBT screw for middle-upper thoracic spine is 5.0 mm, the length is 25 to 35 mm. The caudocephaled angles were 15° to 20°. Due to the small size and transverse angle of middle-upper thoracic pedicle, the CBT screw should be perpendicular to the lamina in the transverse direction (Table 1).

**Table 1:**
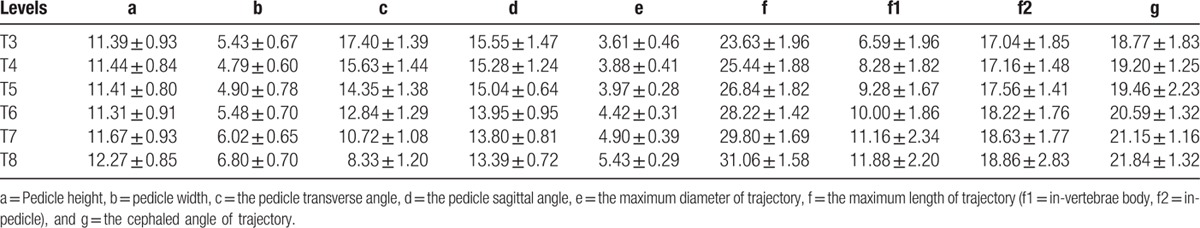
Measurement result from CT scan.

### 3.2 Cadaveric anatomical study

A total of 240 CBT screws were inserted into the middle-upper thoraces. Five screws penetrated the medial or lateral pedicle walls, 2 screws did not fit into the superior endplate of the pedicle, and 5 screws penetrated the superior or inferior pedicle wall. The ratio of the in-body to the in-pedicle trajectory increased from 0.45 ± 0.13 at T3 to 0.78 ± 0.27 at T8. The cephaled angle trajectory exhibited a similar tread. There were no significant differences between the CT scan measurements and the measurements of the cadaveric anatomical study in terms of the ratios of the in-body to the in-pedicle trajectories (T3: *P* = 0.17, T4: *P* = 0.78, T5: *P* = 0.30, T6: *P* = 0.52, T7: *P* = 0.36, T8: *P* = 0.02). In terms of the trajectory cephaled angles, the results of the cadaveric study were below those of the CT scan study (T3: *P* = 0.001, T4: *P* < 0.001, T5: *P* = 0.0007, T6: *P* = 0.0004, T7: *P* < 0.001, T8: *P* < 0.001) (Table 2).

**Table 2:**
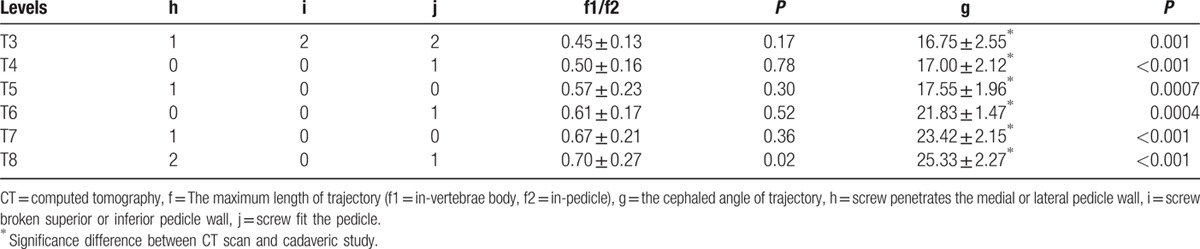
Measurement result from cadaveric specimens and anterior–posterior and lateral films.

## 4 Discussion

As a novel technique, CBT screws demonstrated similar or superior biomechanical characteristics compared with traditional trajectory screws in cadavers with normal bone mineral densities.^[3,17]^ The CBT screws that are inserted through more medial starting points and follow more vertical trajectories exhibit greater pullout loads than traditional pedicle trajectory screws.^[18]^ Santoni et al^[4]^ found that CBT screws and traditional pedicle screws have equivalent pullout strengths and toggle characteristics; however, CBT screws exhibit a 30% increase in pullout load relative to traditional pedicle screws. Perez-Orribo et al^[11]^ reported that there are no significant differences in the mean range of motion or lax zone of CBT screw and traditional pedicle screw fixations during any loading mode. Matsukawa et al^[17]^ reported that CBT screws exhibit 2-fold greater insertional torques than traditional pedicle screws. The use of CBT screws insertion in patients with osteoporosis and loosened screws will represent advancement in the future.

The starting point determined the accuracy of the insert screw.^[19]^ Different pedicle screw techniques have different starting points. For example, the starting point of straight-forward screws located in the midline of the inferior articular facet and upper border of the transverse process,^[20]^ which are utilized in the “in-out-in” technique, employ a far lateral starting point on the posterior cortex of the transverse process.^[21]^ The funnel technique creates a hole in the transverse process as a starting point.^[22,23]^ The morphometrics of thoracic pedicles vary.^[15,24]^ Regarding anatomic variability, the starting point varied across the various levels of the thoracic spine. Several authors recommend the free-hand technique with an exactly defined starting point to achieve an excellent screw position accuracy.^[22,25,26]^ Thus, we chose a starting point based on the free-hand technique as the reference. For T3 to T12, the projection point of the pedicle axis was 4 to 5 mm medial to the lateral margin of the facet and 5 to 8 mm superior to the midline of the transverse process.^[27]^ The CBT screw required medial-to-lateral and caudocephaled angles. The starting point of the CBT screw moved medially and cephalically, which meant less tissue dissection and retraction. The CBT screw technique involved minimally invasive surgeries. Finally, with our starting point, the results revealed a low incidence of pedicle violation (12/240).

In the present study, we found that the maximum trajectory diameter was <5.5 mm, which meant that there was less space for the CBT screw to change direction. Cinotti et al^[28]^ reported that the transverse diameter of the pedicle from T4 through T8 is <5 mm in 48% of cases. Smaller diameter screws could be utilized, but they are associated with an increased risk of breakage. Therefore, the medial-to-lateral angle must be small; thus, we selected an angle perpendicular to the lamina in the transverse direction. After the insertion of the CBT screw, the judgments based on radiographic and cadaveric studies revealed that only 2% (5/240) of the CBT screws penetrated the medial or lateral pedicle walls, and only 2% (5/240) of the CBT screws penetrated the superior or inferior pedicle walls. The pedicle angles inclined more superiorly in the upper and middle thoracic vertebrae.^[29,30]^ We chose a caudocephaled angle of 15° to 20° in the cadaveric study, and this angle was lower than that in the CT study. The results revealed that only 2 screw heads fit the pedicle. The majority of the CBT screws fit the superior endplate but did not target the posterior third of the superior endplate. The use of CBT screws for middle-upper thoracic spine instrumentation is safe and feasible. The surgeons should place the CBT screws under CR monitoring to improve the accuracy and should be familiar with the preoperatively obtained images.

Some limitations of the present study should be mentioned. We used 5.0-diameter CBT screw, which might have reduced the incidence of broken pedicles while increasing the incidence of broken screws. However, base on clinical experience, the limited diameter of pedicle screw was 1 mm wider than pedicle isthmus endosteal.^[31]^ Therefore, the use of a 5.0-diameter screw was also reasonable. Other drawbacks included the lack of postoperative CT scans, which are more accurate than CR. Due to the fact that the measured caudocephaled angle in the cadaveric study was less than that in the CT scan study, the screw length was shorter. The correlation between pullout strength and screw length should be clarified in additional biomechanical studies.

## 5 Conclusions

In conclusion, this study is the first to introduce the application of the CBT screw to the middle-upper thoracic spine and thus provides a theoretical basis for clinical surgery. The use of CBT screw in middle-upper thorax may provide more stability and safety, especially in osteoporotic thoracic and thoracic kyphosis corrections. However, this technique requires further studies to elucidate the biomechanical behavior.
